# Sleepiness, sleep deprivation, quality of life, mental symptoms and perception of academic environment in medical students

**DOI:** 10.1186/s12909-021-02544-8

**Published:** 2021-02-17

**Authors:** Bruno Perotta, Fernanda M. Arantes-Costa, Sylvia C. Enns, Ernesto A. Figueiro-Filho, Helena Paro, Itamar S. Santos, Geraldo Lorenzi-Filho, Milton A. Martins, Patricia Z. Tempski

**Affiliations:** 1 Mackenzie Evangelical School of Medicine – Parana, Curitiba, Brazil; 2 Department of Medicine, School of Medicine of the University of Sao Paulo, Sao Paulo, Brazil; 3 Center for Development of Medical Education, School of Medicine of University of Sao Paulo, Sao Paulo, Brazil; 4 Department of Gynecology & Obstetrics, University of Toronto, Toronto, Canada; 5 Department of Gynecology & Obstetrics, Federal University of Uberlandia, Uberlandia, Brazil; 6 Department of Cardio-Pneumology, School of Medicine of the University of Sao Paulo, Sao Paulo, Brazil

**Keywords:** Medical education, Quality of life, Sleep disorders, Academic environment, Anxiety, Depression, Medical students, Sleep quality, Daytime sleepiness

## Abstract

**Background:**

It has been previously shown that a high percentage of medical students have sleep problems that interfere with academic performance and mental health.

**Methods:**

To study the impact of sleep quality, daytime somnolence, and sleep deprivation on medical students, we analyzed data from a multicenter study with medical students in Brazil (22 medical schools, 1350 randomized medical students). We applied questionnaires of daytime sleepiness, quality of sleep, quality of life, anxiety and depression symptoms and perception of educational environment.

**Results:**

37.8% of medical students presented mild values of daytime sleepiness (Epworth Sleepiness Scale - ESS) and 8.7% presented moderate/severe values. The percentage of female medical students that presented ESS values high or very high was significantly greater than male medical students (*p* <  0.05). Students with lower ESS scores presented significantly greater scores of quality of life and perception of educational environment and lower scores of depression and anxiety symptoms, and these relationships showed a dose-effect pattern. Medical students reporting more sleep deprivation showed significantly greater odds ratios of presenting anxiety and depression symptoms and lower odds of good quality of life or perception of educational environment.

**Conclusions:**

There is a significant association between sleep deprivation and daytime sleepiness with the perception of quality of life and educational environment in medical students.

## Background

Sleep problems are very frequent in the general population and medical students are one group that is vulnerable to poor sleep [1, 2]. The prevalence of sleep disturbances in medical students is higher than in non-medical students [1, 3]. There are many reasons to the high prevalence of sleep problems in medical students, including many hours of classes and study, clinical clerkships that include overnight work, emotional stress, choices concerning lifestyle and many hours using virtual social media [4, 5].

There is evidence that enough good quality sleep is important for long term learning, for neurocognitive and psychomotor performance and for physical and mental health [6]. In addition, sleep deprivation in medical students can make them more vulnerable to depressive and anxiety disorders [1]. Moreover, there are concerns related to patient safety when health professionals are sleep deprived. A review by Curcio et al. suggested that student learning and academic performance are closely related to sleep quantity and quality [7].

To our knowledge there was no previous work that evaluated the relationship between sleep quality and sleep deprivation with quality of life and perception of academic environment in medical students. To better understand the impact of sleep quality and quantity on medical students we analyzed data from a multicenter study with medical students in Brazil [8–11]. The purpose of this study was to evaluate the relationship between sleep deprivation, sleep quality and daytime sleepiness, and quality of life, perception of academic environment and symptoms of depression and anxiety.

## Methods

### Study design and sample

We performed this protocol as part of a multicentric study with 22 Brazilian medical schools (VERAS study, translated to English as “Students’ and Residents’ life in health professions”). Detailed description of this study was previously published [8–11]. Schools participating in the study were from all regions of Brazil, and with a diverse legal status and location (13 public and 9 private, 13 in state capital cities and 9 in other cities). The research protocol was approved by the Ethics Committee of the School of Medicine of the University of Sao Paulo. All medical schools included approved the study.

When our study was performed, Brazil had 153 medical schools with at least one graduating class, with approximately 86,000 medical students. The sample size of the study was defined to enable an effect size of 0.165, with 80% power at a 0.05 significance level, when comparing two samples of equal size. We then increased the sample to 1650 students to account for 30% loss of participants [8–11].

Sixty students were randomly selected from each of 22 medical schools. Five male and five female medical students were selected from each year of the undergraduate program. The selection was performed using a computer-generated list of random numbers [10]. Students were invited to participate by e-mail and social media. Participation was voluntary, without any compensation or incentive. We guaranteed both confidentiality and anonymity, and participating students completed an informed consent form [8–11].

### Data collection

Students accessed an electronic survey platform, that was designed specifically for the study and had 10 days to complete the survey (thirteen questionnaires). After finishing the survey, voluntary received feedback on their scores. Medical students received their score for each domain of each questionnaire and information about the meaning of each result. We offered to the students the opportunity to contact the research group for guidance and/or emotional support. Confidentiality and anonymity were guaranteed in the consent form [8–11].

### Instruments

To assess daytime sleepiness, we used the Epworth Sleepiness Scale (ESS) [12]. This questionnaire consists of 8 self-rated items, each scored from 0 to 3, that measure a subject’s habitual “likelihood of dozing or falling asleep” in common situations of daily living. The final score is the sum of individual items (scores 0–24). Values > 10 are considered excessive daytime sleepiness and values > 15 are considered severe sleepiness. ESS was translated and validated to Brazilian Portuguese [13].

To assess sleep quality, we used Pittsburgh Sleep Quality Index (PSQI) [14]. This questionnaire has 19 items to evaluate subjective sleep quality. We used only the global score of PSQI (range 0 to 21). Higher scores indicate worse sleep quality. Values > 5 are considered poor quality of sleep [14]. PSQI had been previously translated and validated to Brazilian Portuguese [13].

To assess sleep deprivation, we calculated the difference between mean hours of sleep during weekends and mean hours of sleep during weekdays, that was called Sleep Deprivation Index (SDI). SDI was derived from the questions: a) How many hours, on average, did you sleep on weekdays during the last 2 weeks? b) On weekends, if nobody wakes you up, how many hours, on average, do you sleep?

To assess quality of life (QoL) we used three questionnaires:

- WHOQOL-BREF, that has 26 items with four domains: environment, psychological, social relationships, and physical health [15]. Answers are given on a 5-point Likert scale and points within each domain are transformed to a score from 0 to 100. Higher scores represent better QoL (WHOQOL GROUP 1995). This questionnaire was translated and validated to Brazilian Portuguese [16].
- QoL self-assessment, that consisted of two questions to evaluate students’ perception regarding their overall QoL and QoL related to medical school (MSQoL) on a scale from 0 to 10. The items were [1] rate your overall quality of life [2]; rate your quality of life in medical school [8, 10].
- VERAS-Q that is a questionnaire created to evaluate quality of life from students in the health professions. This questionnaire has 45 statements on a 5-points Likert scale divided in four domains (time management, psychological, physical health and learning environment) and a global score [17, 18].

To assess the perception of the educational environment in medical school we used DREEM (Dundee Ready Education Environment Measure), a 50-item questionnaire which evaluates educational environment perceptions. This questionnaire has 5 domains: perceptions of learning, perceptions of teachers, academic self-perceptions, perceptions of atmosphere, and social self-perceptions [19, 20]. Answers are given on a 5-point Likert scale. This questionnaire was translated and validated to Brazilian Portuguese [21].

To assess emotional symptoms, we used Beck Depression Inventory (BDI) and State Trait Anxiety Inventory (STAI). BDI is a 21-item questionnaire to measure depression symptoms [22]. Scores of each item vary from 0 to 3 according to increasing symptom intensity. This questionnaire was translated and validated to Brazilian Portuguese [23]. STAI has a scale with 20 items each evaluating the intensity of state-anxiety and of trait-anxiety symptoms [24]. This questionnaire was also previously translated and validated to Brazilian Portuguese [23].

The results of the reliability analyses performed using the Cronbach’s α coefficient demonstrated that the data had and α value between 0.65 and 0.94 for all domains of the questionnaires (data not shown).

### Statistical analysis

Students were divided according the results of ESS in three groups, respectively ESS ≤ 10, 10 < ESS < 16 and ESS > 16. Comparisons among these three groups were performed using one-way ANOVA followed by Dunn test.

We divided medical students in three groups according to quartiles of sleep deprivation. We present categorical variables as counts and proportions and their distributions across sleep deprivation groups are analyzed using chi-squared trend tests for proportions. Quality of life (Overall, medical school-related, WHOQOL and VERAS-Q), mental symptoms (BDI, STAI-state and STAI-trait), and DREEM scores are presented as medians and interquartile ranges and their distributions across sleep deprivation groups are analyzed using the Jonckheere-Terpstra trend test. We built binary logistic regression models to study the association between sleep deprivation and daytime sleepiness, and the association between sleep deprivation and high scores in each of these scales. High scores were defined as a score equal or above the median for the whole sample. Binary logistic models are presented adjusted for age, sex, and year of medical school. Significance level was set at 0.05. Analyses were performed using R software, version 3.2.0.

## Results

As previously shown, of 1650 randomly selected students, 1350 (81.8%) accepted to participate and completed the study [8–11]. The main reason to refuse to participate in the study (16.6%) was lack of time. Their ages ranged between 17 and 40 (22.8 ± 1.3) years old.

From the 1350 participants, 714 (52.9%) were women, 459 (34.0%) were in the 1st or 2nd year of medical school (basic sciences), 491 (36.4%) were in the 3rd or 4th year of medical school (clinical sciences) and 400 (29.6%) in the last 2 years of medical course (clerkships).

Table 1 shows the results of Epworth Daytime Sleepiness Scale (ESS): 37;8% medical students presented high values of ESS and 8.7% presented very high values. The percentages of female medical students that presented ESS values high or very high were significantly greater than male medical students.

**Table 1:** Results of the Epworth Daytime Sleepiness Scale (ESS) in all medical students evaluated

ESS results	Males	Females	Total
0–10	388 (61.0%)	334 (46.8%)*	722 (53.5%)
11–15	211 (33.2%)	299 (41.9%)*	510 (37.8%)
16–24	37 (5.8%)	81 (11.3%)*	118 (8.7%)

* *P* <  0.05 compared to males

**Fig. 1:**
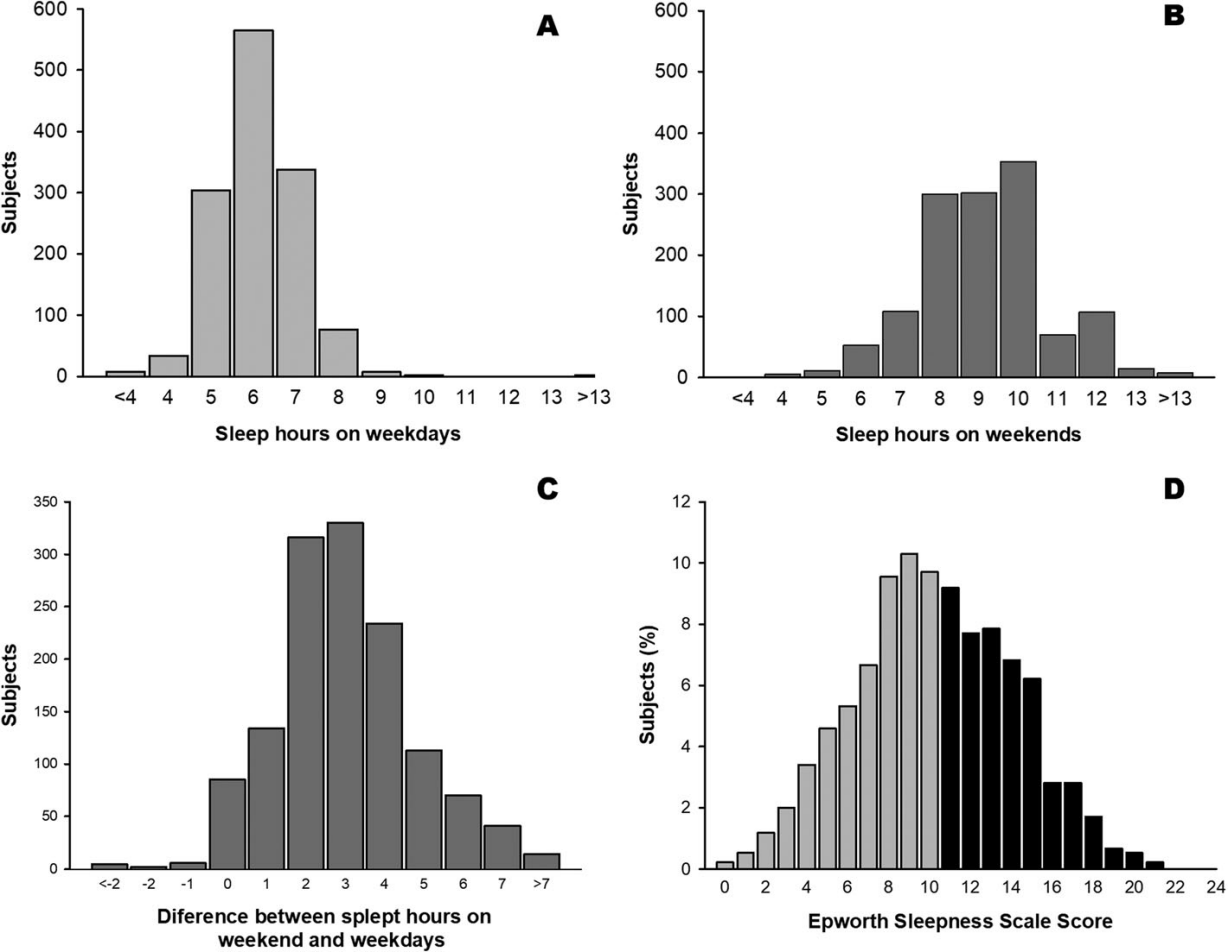
Sleep pattern and daily sleepiness among medical students. Histograms represent the distribution of self-related sleep hours during weekdays (**a**), weekends (**b**), difference between mean weekend and weekday sleep hours (**c**) and Epworth Sleepiness Scale (ESS) scores of medical students (**d**). Gray bars represent normal values of ESS and black bars represent increased daytime somnolence

Figure 1 shows the distribution of self-related sleep hours during weekdays (A), weekends (B), difference between mean weekend and weekday sleep hours (C) and ESS scores of medical students (D).

We evaluated the differences in the results of questionnaires of quality of life, education environment, and depression and anxiety symptoms among medical students with normal values of ESS (< 10), students with values between 11 and 15 and students with values > 15.

**Fig. 2:**
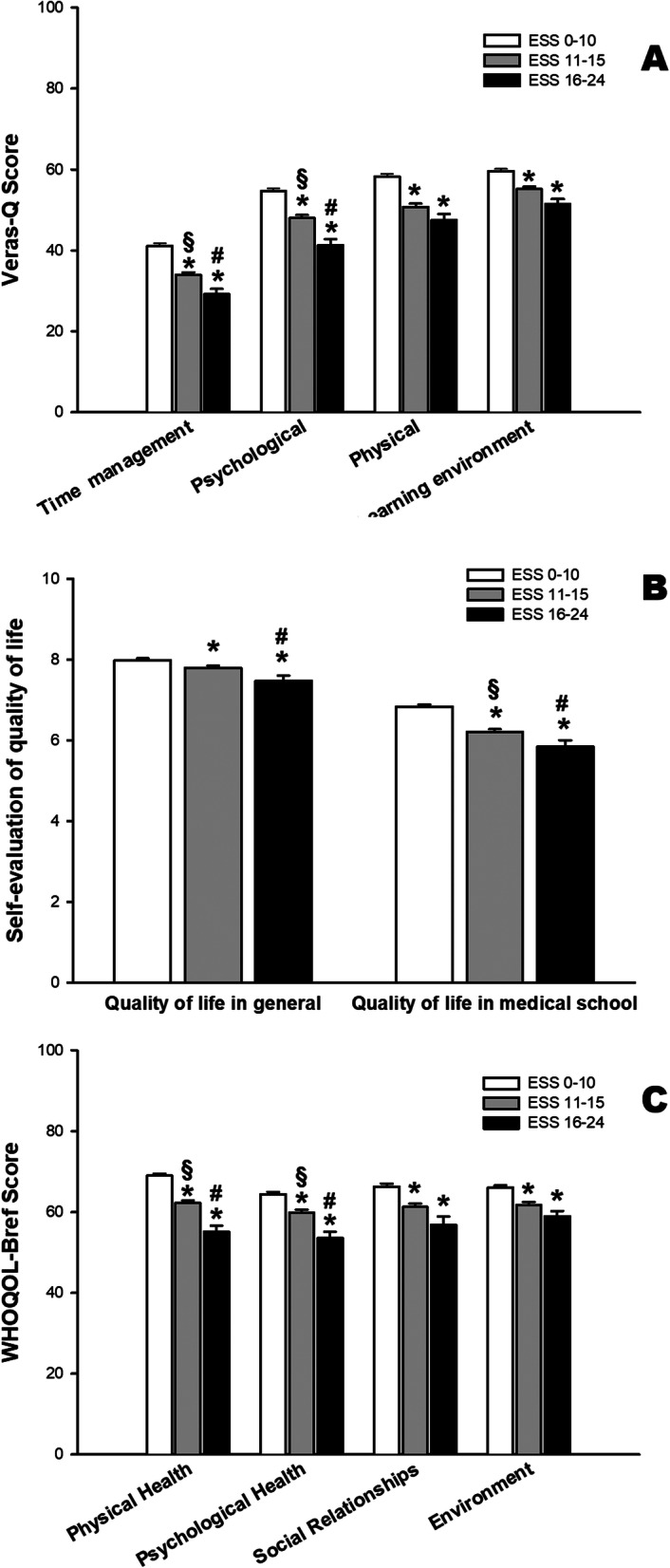
Quality of life of medical students decreases with higher daily sleepiness scores. Mean and standard error values of VERAS-Q (**a**), self-evaluation of QoL (**b**) and WHOQOL-BREF questionnaires (**c**) in the three groups of medical students based on ESS scores. * *p* <  0.05 compared to ESS 0–10; § p <  0.05 compared to ESS 16–24; # *p* < 0.05 compared to ESS 11–15

The association between excessive daytime sleepiness and quality of life is shown in Fig. 2. We observed a dose-effect pattern, with lower values of ESS corresponding to higher values of quality of life scores. We observed statistically significant differences among the three groups in all domains of WHOQOL-BREF and VERAS-Q questionnaires and in the scores of quality of life in general and medical school-related quality of life.

**Fig. 3:**
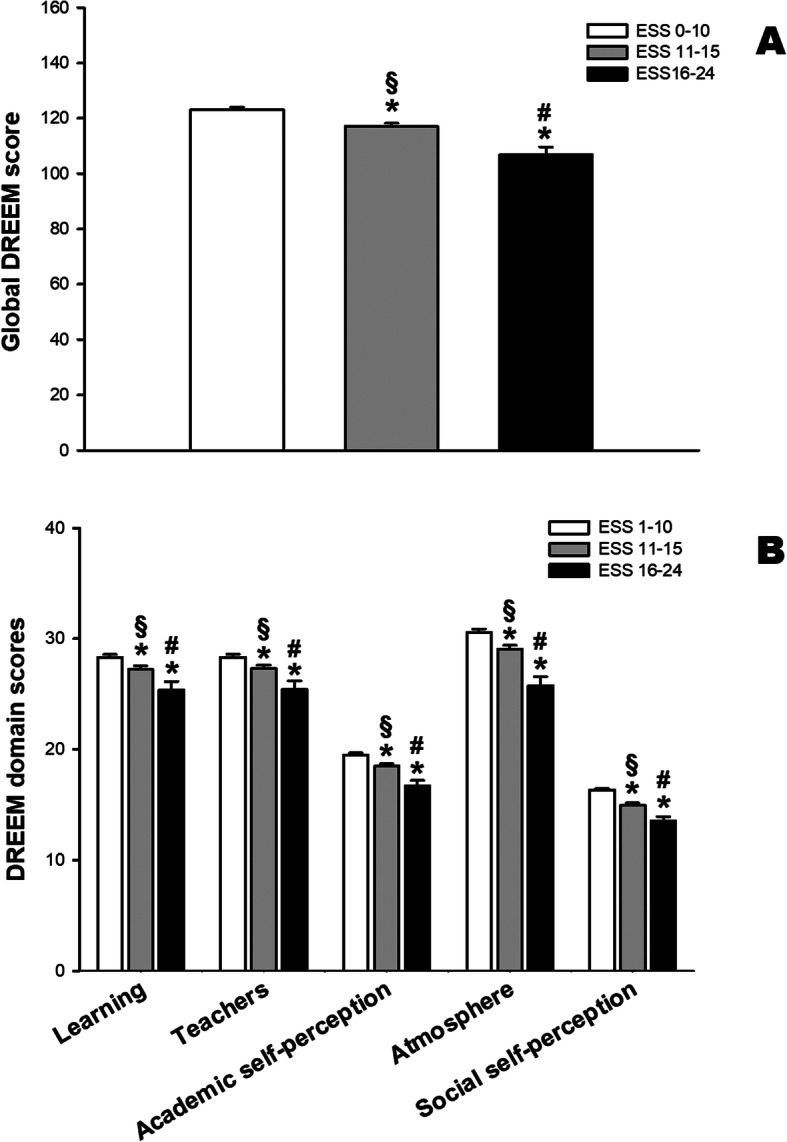
Medical students that presents higher daily sleepiness scores (ESS) showed lower perception of educational environment. Bars indicate mean (and standard error) values of DREEM global (**a**) and domain scores (**b**) * *p* < 0.05 compared to ESS 0–10; § *p* < 0.05 compared to ESS 16–24; # p < 0.05 compared to ESS 11–15

We also observed that students with higher values of ESS presented a worse perception of education environment. Both in global DREEM score and in the five domains of DREEM there were statistically significant differences among the three groups of medical students concerning the results of ESS (Fig. 3).

**Fig. 4:**
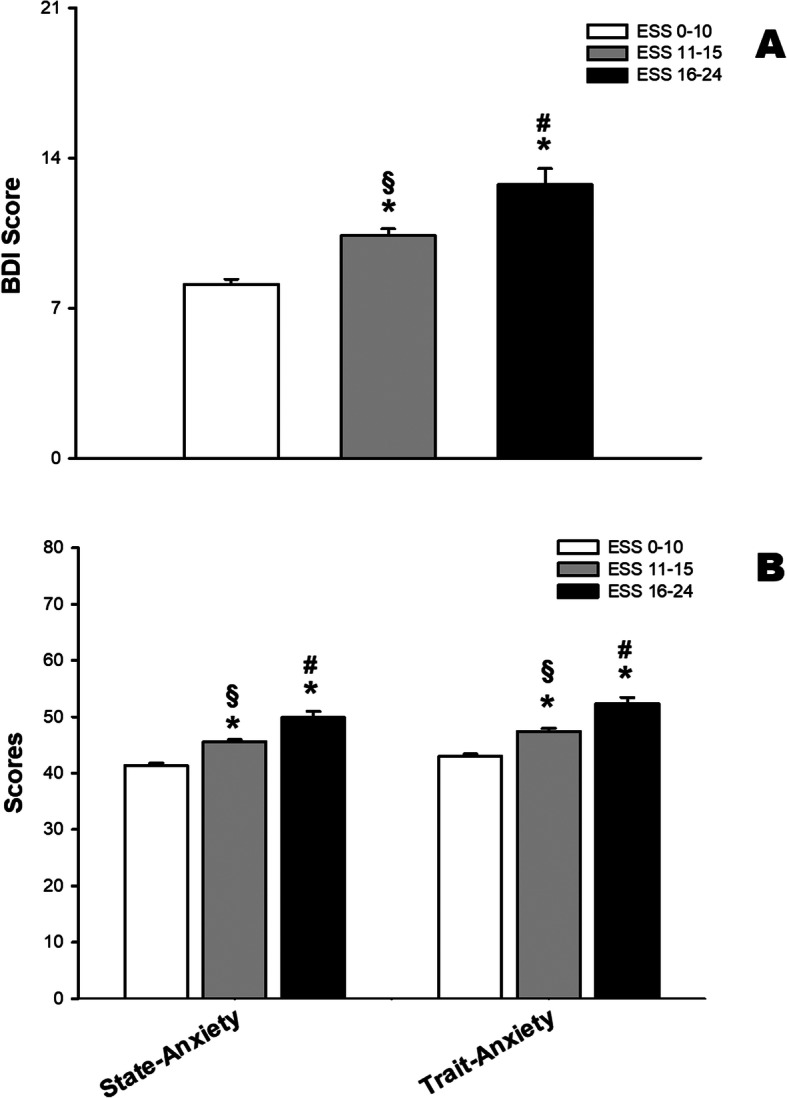
Higher scores of sleepiness are associated with higher scores of depression and anxiety. Beck Depression Inventory (BDI (**a**) and State and Trait Anxiety scores (**b**) (Means and standard errors). BDI scores range from 0 to 21 and STAI scores range from 20 to 80. * p < 0.05 compared to ESS 0–10; § p < 0.05 compared to ESS 16–24; # p < 0.05 compared to ESS 11–15

Higher scores of daytime sleepiness were also associated with higher scores of depression symptoms and with state and trait anxiety scores. We also observed a dose-response relationship and the differences were statistically significant among the three groups of ESS values (Fig. 4).

**Fig. 5:**
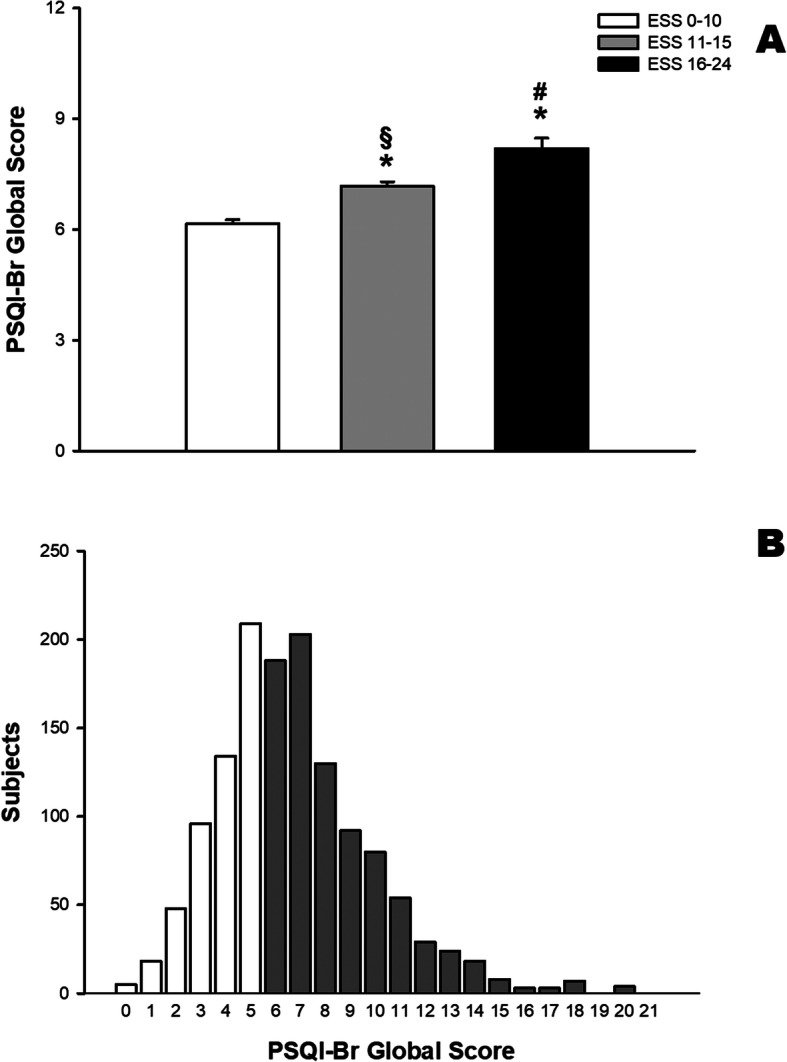
Medical students that presents higher daily sleepiness score showed lower quality of sleep measured by PSQI-Br. PSQI-Br global score ranges from 0 to 21, and lower scores represent better quality of sleep. **a** Mean (and standard error) values of PSQI-Br global scores. **b** Distribution of number of medical students with each value of PSQI-Br. Values higher than 5 indicate poor quality of sleep (gray bars). * *p* < 0.05 compared to ESS 0–10; § p < 0.05 compared to ESS 16–24; # p < 0.05 compared to ESS 11–15

Medical students that presented higher ESS scores showed lower quality of sleep measured by PSQI. PSQI global score range from 0 to 21, lower scores represent better quality of sleep (Fig. 5a). Figure 5b shows the distribution of PSQI scores in all medical students.

We divided medical students in four quartiles concerning the values of this sleep deprivation index (SDI), with SDI respectively ≤2 (Q1), =3 (Q2), =4 (Q3) and >  4 (Q4) hours. Table 2 shows the distribution of medical students and values of the studied questionnaires according to deprivation groups (Q1, Q2 + Q3 and Q4).

**Table 2:** Description of the study sample, according to sleep deprivation groups, from Sleep Deprivation Index

	Q1 (*N* = 536) ≤ 2 h	Q2-Q3 (*N* = 564) 3 and 4 h	Q4 (*N* = 238) > 4 h	Total (*N* = 1338)
Age (mean ± SD)	23.0 ± 3.0	22.5 ± 2.9	22.6 ± 3.0	22.7 ± 3.0
Year of medical school (N (%))				
1st/2nd (Basic)	175 (32.6%)	196 (34.8%)	83 (34.9%)	454 (33.9%)
3rd/4th (Clinical)	195 (36.4%)	209 (37.1%)	81 (34.0%)	485 (36.2%)
5th/6th (Clerkship)	166 (31.0%)	159 (28.2%)	74 (31.1%)	399 (29.8%)
Female sex - N (%)	276 (51.5%)	304 (53.9%)	131 (55.0%)	711 (53.1%)
WHOQOL (median [P25 - P75])				
Physical	**71.4 [60.7–78.6]**	**64.3 [57.1–75.0]**	**60.7 [46.4–71.4]**	67.9 [53.6–75.0]
Psychological	**66.7 [54.2–75.0]**	**62.5 [54.2–70.8]**	**58.3 [45.8–66.7]**	62.5 [54.2–75.0]
Social Relationships	**66.7 [58.3–75.0]**	**66.7 [50.0–75.0]**	**58.3 [50.0–75.0]**	66.7 [50.0–75.0]
Environment	**65.6 [56.3–75.0]**	**65.6 [53.1–75.0]**	**59.4 [50.0–68.8]**	65.6 [53.1–75.0]
VERAS-Q (median [P25 - P75])				
Time use	**39.8 [29.6–52.3]**	**36.4 [25.0–45.5]**	**29.6 [20.4–40.9]**	36.4 [25.0–47.7]
Psychological	**54.2 [43.8–66.7]**	**50.0 [39.6–60.4]**	**43.8 [33.3–54.2]**	50.0 [39.6–62.5]
Physical Environment	**59.4 [43.8–71.9]** **58.9 [50.0–67.9]**	**53.1 [40.6–65.6]** **57.1 [49.5–66.1]**	**46.9 [34.4–59.4]** **55.4 [46.4–62.5]**	53.1 [40.6–68.8] 57.1 [48.2–66.1]
Quality of life (median [P25 - P75])				
Overall	8.0 [7.0–9.0]	8.0 [7.0–9.0]	8.0 [7.0–9.0]	8.0 [7.0–9.0]
Medical school-related	**7.0 [6.0–8.0]**	**7.0 [6.0–7.0]**	**6.0 [5.0–7.0]**	7.0 [6.0–8.0]
Mental symptoms (median [P25 - P75])				
Depression (BDI)	**7.0 [3.0–11.0]**	**8.0 [5.0–13.0]**	**11.0 [7.0–17.0]**	8.0 [4.0–13.0]
Anxiety-state	**41.0 [34.0–50.0]**	**42.0 [35.0–51.0]**	**47.0 [40.0–57.0]**	43.0 [35.0–52.0]
Anxiety-trait	**43.0 [34.0–50.0]**	**46.0 [37.8–53.0]**	**50.0 [41.2–59.0]**	45.0 [37.0–53.0]
DREEM (median [P25 - P75])				
Perceptions of learning	**28.5 [23.0–33.0]**	**28.0 [23.0–33.0]**	**26.0 [22.0–32.0]**	28.0 [23.0–33.0]
Perception of teachers	28.0 [23.0–32.0]	28.0 [24.0–33.0]	27.0 [23.0–31.0]	28.0 [23.0–32.0]
Perceptions of the atmosphere	**20.0 [16.0–23.0]**	**19.0 [16.0–23.0]**	**17.5 [14.2–21.0]**	19.0 [15.2–22.0]
Academic self-perceptions	**31.0 [25.0–36.0]**	**30.5 [25.0–35.0]**	**28.0 [23.0–33.0]**	30.0 [25.0–35.0]
Social self-perceptions	**16.0 [13.0–20.0]**	**16.0 [13.0–18.2]**	**14.0 [11.0–17.0]**	16.0 [13.0–19.0]
Global	**123.5 [102.8–142.0]**	**121.0 [101.8–139.0]**	**113.0 [98.0–128.8]**	120.0 [101.0–139.0]

Groups that showed statistically significant differences (*P* < 0.05) are in bold

Table 3 shows the results of binary logistic regression models. We show the odds ratios (and 95% confidence intervals) for the association between sleep deprivation groups and high quality of life, depression and anxiety symptoms and perception of academic environment. The results are presented crude and adjusted for age, sex and year of medical school. Group Q1 was used as reference and the odds ratio that were statistically significant are presented in bold.

**Table 3:** Odds ratios (and 95% confidence intervals) for the association between sleep deprivation groups and high quality of life, mental symptoms, and DREEM scores

	Crude			Adjusted		
	Q1 ≤ 2 h	Q2-Q3 3 and 4 h	Q4 > 4 h	Q1 ≤ 2 h	Q2-Q3 3 and 4 h	Q4 > 4 h
WHOQOL						
Physical	Ref (1.0)	**0.61 (0.48–0.77)**	**0.36 (0.26–0.49)**	Ref (1.0)	**0.61 (0.48–0.78)**	**0.36 (0.26–0.49)**
Psychological	Ref (1.0)	**0.63 (0.49–0.80)**	**0.41 (0.30–0.55)**	Ref (1.0)	**0.62 (0.49–0.80)**	**0.41 (0.30–0.55)** **0.56 (0.41–0.77)**
Social Relationships	Ref (1.0)	**0.70 (0.55–0.89)**	**0.58 (0.42–0.78)**	Ref (1.0)	**0.68 (0.54–0.87)**	
Environment	Ref (1.0)	0.88 (0.69–1.11)	**0.45 (0.33–0.62)**	Ref (1.0)	0.84 (0.66–1.07)	**0.44 (0.32–0.60)**
VERAS-Q						
Time use	Ref (1.0)	**0.69 (0.54–0.87)**	**0.37 (0.27–0.50)**	Ref (1.0)	**0.68 (0.54–0.87)**	**0.36 (0.27–0.50)**
Psychological	Ref (1.0)	**0.58 (0.46–0.74)**	**0.44 (0.32–0.60)**	Ref (1.0)	**0.58 (0.45–0.74)**	**0.44 (0.32–0.60)**
Physical	Ref (1.0)	**0.71 (0.56–0.90)**	**0.38 (0.28–0.52)**	Ref (1.0)	**0.69 (0.54–0.88)**	**0.37 (0.27–0.51)**
Environment	Ref (1.0)	0.85 (0.67–1.07)	**0.65 (0.48–0.88)**	Ref (1.0)	0.81 (0.63–1.03)	**0.62 (0.46–0.85)**
Quality of life						
Overall	Ref (1.0)	0.97 (0.75–1.25)	0.76 (0.55–1.04)	Ref (1.0)	0.94 (0.73–1.21)	0.74 (0.54–1.01)
Medical school-related	Ref (1.0)	**0.73 (0.58–0.93)**	**0.45 (0.33–0.62)**	Ref (1.0)	**0.71 (0.56–0.91)**	**0.44 (0.32–0.60)**
Mental symptoms						
BDI	Ref (1.0)	**1.47 (1.16–1.86)**	**2.99 (2.16–4.14)**	Ref (1.0)	**1.46 (1.15–1.86)**	**3.01 (2.16–4.19)**
Anxiety-state	Ref (1.0)	1.07 (0.85–1.36)	**1.95 (1.42–2.67)**	Ref (1.0)	1.07 (0.84–1.36)	**1.94 (1.42–2.67)**
Anxiety-trait	Ref (1.0)	**1.56 (1.23–1.98)**	**2.62 (1.90–3.60)**	Ref (1.0)	**1.55 (1.22–1.98)**	**2.63 (1.90–3.64)**
DREEM						
Perceptions of learning	Ref (1.0)	0.81 (0.64–1.02)	**0.63 (0.46–0.86)**	Ref (1.0)	**0.79 (0.62–0.9998)**	**0.62 (0.45–0.84)**
Perception of teachers	Ref (1.0)	1.07 (0.84–1.36)	**0.71 (0.53–0.97)**	Ref (1.0)	1.04 (0.81–1.32)	**0.70 (0.51–0.96)**
Perceptions of atmosphere	Ref (1.0)	0.80 (0.63–1.02)	**0.46 (0.34–0.63)**	Ref (1.0)	0.80 (0.63–1.02)	**0.46 (0.34–0.63)**
Academic self-perceptions	Ref (1.0)	0.92 (0.73–1.17)	**0.53 (0.39–0.73)**	Ref (1.0)	0.91 (0.72–1.16)	**0.53 (0.39–0.72)**
Social self-perceptions	Ref (1.0)	**0.72 (0.57–0.91)**	**0.46 (0.33–0.62)**	Ref (1.0)	**0.71 (0.56–0.90)**	**0.45 (0.33–0.62)**
Global	Ref (1.0)	0.90 (0.71–1.14)	**0.44 (0.32–0.61)**	Ref (1.0)	0.89 (0.70–1.13)	**0.44 (0.32–0.60)**

High scores are defined as those equal of above the median for the whole sample. *P*-values below 0.05 are in bold. Adjusted models are adjusted for age, sex and year of medical school

Groups with higher sleep deprivation (Q2 + Q3 and Q4) had lower odds for higher scores of quality of life in all domains of VERAS-Q and WHOQOL-BREF questionnaires with the exception of environment domains of group Q2 + Q3. Interestingly, lower odds for quality of life were observed in the groups with sleep deprivation only for medical school-related quality of live but not for overall QoL.

We observed higher odds for depression symptoms in medical students with higher differences between weekends and weekdays sleep hours (SDI). Medical student that reported more than 4 h of SDI had an odds ratio of 3.01 (2.16 to 4.19) of higher depression symptoms compared to students with a SDI less than 3. We also observed higher odds rations of higher anxiety symptoms for state anxiety in Group Q4 and for trait anxiety in groups Q2 + Q3 and Q4.

When we studied the odds ratios of higher DREEM scores, we observed statistically significant lower odds ratios in Group Q4 compared to Q1 in global DREEM scores and in all DREEM domains (learning, teachers, educational atmosphere, academic and social self-perception). Group Q2 + Q3 presented lower odds only in two domains (perception of learning and social self-perception).

Table 4 shows the results of a binary logistic regression model for the association between sleep deprivation index (SDI) and daytime sleepiness (ESS). We show the odds ratios (and 95% confidence intervals) for the association between sleep deprivation groups and daytime sleepiness. The results were adjusted for age, sex and year of medical school. Group Q1 was used as reference. Students in the quartile 2 and quartile 3 of the SDI had an increase of 59,9% the odds of having pathologic values of daytime sleepiness, in comparison with Q1. In addition, students in the quartile 4 of SDI had an increase of 122,8% in the odds of having pathologic values of daytime sleepiness, in comparison with Q1 group.

**Table 4:** Results of binary logistic regression models for the association between sleep deprivation index (SDI) and Epworth scale (daytime sleepiness)

	SDI	Adjusted data OR (95% CI)	P
Epworth > 10	Q1 (≤ 2 h)	Ref (1.0)	
	Q2 + Q3 (3 and 4 h)	**1.56 (1.25 to 2.04)**	< 0.001
	Q4 (≥ 4 h)	**2.23 (1.62 to 3.05)**	< 0.001

## Discussion

Our data reveal consistent associations between daytime sleepiness and sleep deprivation and worse perception of quality of life and academic environment, and anxiety and depression symptoms in medical students. A dose-response relationship was observed for these associations.

In our study, there was a high frequency of students who had high scores on the Epworth scale (46.5%). This number, if compared with most studies involving medical students, was impressive. A study from Malaysia showed a percentage of 35.0% [25] of high scores on the Epworth scale. In India, this value was 30.6% [26]. Our data also showed that females had greater daytime sleepiness in relation to the males.

Our results showed that there was also a high percentage of students who had poor sleep quality by PSQI (62.2%). This number was higher than other studies in medical students, with scores ranging from 19.0% in China [27], 38.9% in Brazil [28] and 40.0% in Lithuania [29]. A national study, which evaluated the general adult population, showed a mean of 4.9 of the overall PSQI score and worse scores in females [30]. Our data did not show differences between males and females, and we observed a worse mean of the overall PSQI score.

Some studies have evaluated sleep in healthy young general population, identifying habitual sleep ranges from 7.0 to 8.5 h, and their determinants are social factors and lifestyle [31–34]. However, when offered the opportunity of extended sleep time in experiments with protected hours, the amount of nocturnal sleep can increase more than 1 h, ranging from 8.4 to 8.9 h [31–36]. The recommendation of the National Sleep Foundation is that individuals from 18 to 25 years of age sleep between 7 and 9 h [37]. The extended period of sleep brings potential benefits to the individual because this implies that all phases of sleep are respected, allowing physical and mental restoration [32]. One practical way in which people compensate for the lack of sleep that may incorporate into their routine is a short nap throughout the day.

The difference between the hours of sleep in the week and at the weekend associated with not meeting the actual need for sleep suggests that many students in our study had chronic sleep deprivation. The smaller mean hours of sleep during the week in the group with worse daytime sleepiness scores (Epworth> 10) also reinforce this data. Other studies have shown that young adults have sleep deprivation from one to three hours at night during the week, with a much longer sleep duration and wake-up time later at weekends [38]. Coupled with this behavior, many medical students view sleep deprivation as a symbol of dedication to the profession [39]. This aspect has a strong influence of the hidden curriculum, which concerns the student’s socialization in the process of becoming a doctor, or the construction of their professional identity, acquiring habits and behaviors patterns of their peers and models [40]. The common sense is that the successful doctor is the one who is too busy to abstain from hours of leisure, socializing and self-care, in favor of the health care of others [41]. This model that underestimates self-care can be assimilated and reproduced by students, sacrificing their hours of sleep for other interests.

Specialists in time management suggest that the agenda begins by delimiting the necessary hours of sleep and from there the other daily tasks are distributed. The question that arises is that there is a desire among the students to include all complementary training opportunities to the formal curriculum, often causing harm to their health. This overload can be motivated both by the competitiveness among the students and by the generational multitasking characteristic [42].

Few data exist on the medical student’s routine in the past. A 1968 study in England found that on average the medical student slept eight hours a day and that the amount of sleep did not change between the week and the weekend [43]. An Australian study reported the worst academic performance when waking later in the morning, especially at weekends [44]. The same author, years later, after developing the Epworth scale, found an average of this score of 7.6 [45], whereas in our data the average daytime sleepiness score was 10.3. The analysis of these studies shows that in addition to the cultural differences, it is necessary to highlight the historicity of the samples.

Some authors compared the sleep of medical students with that of other courses. There is a large percentage of college students in general who sleep less than 7 h per night, ranging from 24 to 49% [46]. Medical students had worse PSQI scores in relation to Law and Economics courses in Lithuania [29].

Several studies have reported the relationship between daytime sleepiness and academic performance. There were better performances in students who slept earlier and who had greater hours of sleep during the week. Sleep deprivation has negative effects on emotional intelligence, including the ability to demonstrate empathy [47–49]. Of course, these studies report only associations, and cause-effect of sleepiness versus academic performance or emotional abilities cannot be precisely established.

In the same context, it is unclear whether sleepiness leads to deterioration of the student’s mental health, or whether drowsiness can be one of the consequences of anxiety or depression. A national study revealed an increased risk of minor psychiatric disorders among students with sleepiness, sleep interruption, insomnia, and sleep hours of less than 7 h [50]. Loayza et al. [50] suggest that the evaluation of sleepiness in medical students can be a good tool for psychiatric screening and preventive measures.

The overall PSQI scores were related to the range of ESS scores, that is, there was a positive association of the instruments, indicating that the higher the PSQI Global score (meaning poorer sleep quality), the greater the tendency of the individual have an ESS altered score (indicating greater daytime sleepiness).

Few studies compared WHOQOL-BREF with Epworth scale, and these studies were from specific populations, such as elderly patients with chronic pain or sleep apnea [51–53]. All studies revealed a relationship between sleepiness and decreased the quality of life.

Our DREEM results show that students had a more positive than negative perception of educational environment (total score between 101 and 150), according to the syntax of DREEM [19]. The mean of the global score was similar to the results of other studies conducted in developing countries such as Iran, India, Kuwait and Sri Lanka [54–57].

Odds Ratio (OR) values were significant for most associations between sleep and quality of life and educational environment. These logistic regression results are robust because they carefully exclude confounding factors such as age, sex, and course year. With this analysis, the impact of sleep deprivation on the medical student’s quality of life confirms the practical relevance of this issue. However, data on quality of life are multifactorial and sleepiness is not an isolated factor in the worsening of the quality of life and in the perception of the educational environment. It is worth mentioning that only the group with the highest drowsiness (Epworth quartile 4) presented a significant association of ORs for the domains of DREEM and Global score, except for perception of learning and social relation’s domains, which also showed significance in the intermediate drowsiness group (quartiles 2 + 3).

The present study has some strengths: the original format, the national multicenter design, with an expressive number of randomized respondents, a low number of losses, a high response rate and a variety of instruments that analyze the quality of life, sleep, emotional symptoms, and medical student educational environment. Another positive aspect of the study was the possibility for respondents to receive feedback on their results and the opportunity for support and guidance.

Our study has as limitations the transversal design that does not allow us to analyze causality and the fact that the results are generalizable only to the universe of Brazilian students, although we can infer that they are similar to those found in other cultures. There are some limitations of studies that use self-reports. Specifically, in relation to studies of sleep, the results can be compared with more objective measures, such as polysomnography or actigraphy. More stressed individuals tend to report more sleepiness and fatigue in relation to people who are less stressed [58]. Concerning quality of life, individuals with more critical views may negatively direct their responses to some items.

## Conclusions

Sleep deprivation and daytime sleepiness are associated to a worse the perception of quality of life and educational environment and depression and anxiety symptoms in medical students.

Curricular changes that include redistribution of academic activities, individual orientation for mentoring activity, health promotion programs and protected hours for study and leisure are valid strategies to assist the student in the management of his/her time, which indirectly can improve his / her learning, sleep and decrease their daytime sleepiness, ultimately improving the medical student’s quality of life.

## Data Availability

The datasets used and/or analysed during the current study are available from the corresponding author on reasonable request.

## Abbreviations

ESS: Epworth Sleepiness Scale
PSQI: Pittsburgh Sleep Quality Index
SDI: Sleep Deprivation Index
QoL: Overall quality of life
MSQoL: Medical School related quality of life
BDI: Beck Depression Inventory
STAI: State Trait Anxiety Inventory
DREEM: Dundee Ready Education Environment Measure
VERAS-Q: Questionnaire to evaluate quality of life in students of health professions
